# Nature versus nurture: Structural equation modeling indicates that parental care does not mitigate consequences of poor environmental conditions in Eastern Bluebirds (*Sialia sialis*)

**DOI:** 10.1002/ece3.8207

**Published:** 2021-10-20

**Authors:** Madeline Sudnick, Bekka S. Brodie, Kelly A. Williams

**Affiliations:** ^1^ Honors Tutorial College Ohio University Athens Ohio USA; ^2^ Department of Biological Sciences Ohio University Athens Ohio USA; ^3^ Ohio Center for Ecology and Evolutionary Studies Ohio University Athens Ohio USA

**Keywords:** Eastern Bluebird (*Sialia sialis*), ectoparasites, food availability, growth rate, hematocrit, offspring development, parental behaviors, structural equation modeling

## Abstract

How organisms respond to variation in environmental conditions and whether behavioral responses can mitigate negative consequences on growth, condition, and other fitness measures are critical to our ability to conserve populations in changing environments. Offspring development is affected by environmental conditions and parental care behavior. When adverse environmental conditions are present, parents may alter behaviors to mitigate the impacts of poor environmental conditions on offspring. We determined whether parental behavior (provisioning rates, attentiveness, and nest temperature) varied in relation to environmental conditions (e.g., food availability and ectoparasites) and whether parental behavior mitigated negative consequences of the environment on their offspring in Eastern Bluebirds (*Sialia sialis*). We found that offspring on territories with lower food availability had higher hematocrit, and when bird blow flies (*Protocalliphora* spp.) were present, growth rates were reduced. Parents increased provisioning and nest attendance in response to increased food availability but did not alter behavior in response to parasitism by blow flies. While parents altered behavior in response to resource availability, parents were unable to override the direct effects of negative environmental conditions on offspring growth and hematocrit. Our work highlights the importance of the environment on offspring development and suggests that parents may not be able to sufficiently alter behavior to ameliorate challenging environmental conditions.

## Graphical abstract

We found that while parents altered their behavior in response to resource availability, they did not respond to parasite presence. Parents were unable to compensate for the negative effects of low‐resource availability and avian blow flies on offspring growth and condition.

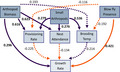

## 1 INTRODUCTION

Growth and condition during development not only are linked to adult phenotype, reproduction, and survival in a variety of taxa including birds (Lindström, 1999), mammals (Bowen et al., 2015), salamanders (Krause et al., 2011), and lizards (Uller & Olsson, 2010) but are also affected by early environmental conditions (Beltrán et al., 2020; Hart et al., 2006; Pérez et al., 2016; Ronget et al., 2018). Offspring development can also be impacted by parental behavior (Eggert et al., 1998; Grew et al., 2019; Hepp et al., 2006; Klug & Bonsall, 2014). Organisms may alter behavior in response to environmental conditions (Brinkerhoff et al., 2005; Sih et al., 2010; Tuomainen & Candolin, 2011) mitigating negative impacts of environmental variation (Fey et al., 2019; Laux et al., 2016; Tripet & Richner, 1997). Therefore, determining how parents respond to environmental variation and whether parental behavior buffers offspring from direct health consequences due to poor environments is critical to understanding recruitment and demographic parameters.

Offspring may face trade‐offs between growth and condition (e.g., residual body mass and hematocrit) when adverse environmental conditions are present. Condition during development can have carryover effects into adulthood leading to reduced reproduction (reviewed in Lindström, 1999) and affect the habitat quality adults occupy (Verhulst et al., 1997). Food deserts are areas with low food availability and are associated with adverse health outcomes in humans (reviewed in Gundersen & Ziliak, 2015; Walker et al., 2010). In birds, low food availability (e.g., arthropod abundance) is associated with reduced nestling hematocrit (packed red blood cell volume, a common measure of condition; Hoi‐Leitner et al., 2001) and offspring growth is slowed (Emlen et al., 1991; McKinnon et al., 2012; Numata et al., 2004). Additionally, if the quality of available prey is low, offspring may need greater quantities of food in order to meet nutritional requirements (Wright et al., 1998) and the size of prey delivered to offspring must match offspring size (Wiebe & Slagsvold, 2014). Therefore, both the overall biomass and the type of prey in the environment may impact offspring development.

Ectoparasites including feather‐ or blood‐feeding mites, lice, and avian blow flies can have negative impacts on hosts (Deeming & Reynolds, 2015). Bird blow flies (*Protocalliphora* spp.; blow flies) are ectoparasites that feed on offspring blood, negatively affect growth and condition (Merino & Potti, 1995), and affect how resources are allocated (O’Brien & Dawson, 2008). Higher ectoparasite loads are associated with decreased body mass (Brown & Brown, 1986), decreased hematocrit (Potti et al., 1999; Simon et al., 2005), and slower feather growth (Brommer et al., 2011). However, trade‐offs between growth and condition may occur with individuals prioritizing growth at the expense of condition. For example, Barn Swallow (*Hirundo rustica*) nestlings exposed to increased parasite infestation had faster feather growth but were in poorer condition than nests with fewer parasites (Saino et al., 1998).

Parent birds of altricial young provide food to nestlings, brood young to maintain nest temperatures, and engage in nest maintenance behaviors. Provisioning rates and nest attendance are positively correlated with food availability and nest temperature (Ardia et al., 2009; Rauter et al., 2000). Increased nest attendance and provisioning can directly improve growth rates and condition (Ardia, 2013; Hopkins et al., 2011; Nord & Nilsson, 2011; Potti et al., 1999). The environment can also indirectly influence growth rate and condition through alteration of parental care behaviors. In blue tits (*Cyanistes caeruleus*), parents increased food delivery to parasitized nests, and parasitized offspring did not suffer reduced body condition (Tripet et al., 2002; Tripet & Richner, 1997). In environments with low arthropod availability, parents can increase foraging time or distance to meet the nutritional needs and maintain development of offspring (Tremblay et al., 2005). However, in some species, parents are not able to effectively change behavior to compensate for the effects of environmental conditions on offspring, resulting in negative developmental consequences for offspring (Cantarero et al., 2013).

Determining whether and how environmental conditions affect parental behaviors and impact offspring development will provide insight for understanding the influences on offspring growth and condition in variable habitats. Eastern Bluebirds (*Sialia sialis*) are cavity nesting birds that use artificial nest cavities (e.g., wooden bird boxes), and are tolerant of human observation during breeding, and while only the female incubates eggs, both parents provision young and participate in nest maintenance and defense (Gowaty & Plissner, 2020) making this species a model system to understand how environmental conditions and biparental care affect offspring development. Adults provision young with a wide range of arthropod prey including adult and larval Lepidoptera (moths and butterflies), Araneae (spiders), Orthopterans (crickets, grasshoppers, and katydids), Coleoptera (beetles), and other small prey items (Gowaty & Plissner, 2020). Adults capture arthropod prey that averages 13 mm in length, with 42% of prey fed to young measuring <13 mm (Goldman, 1975), suggesting that small arthropods are an important food resource for offspring.

In this study, we tested the hypotheses that environmental factors (i.e., food availability and ectoparasites) and parental care behaviors (i.e., parental attentiveness, provisioning rate, and nest temperature) impact growth rate (i.e., tarsus) and hematocrit of Eastern Bluebird nestlings. We used general linear mixed‐effects models to test the direct effects of these factors on offspring condition. We predicted that increased parental care would be directly associated with increased nestling growth rate and hematocrit, while low arthropod availability and ectoparasites would be associated with reduced growth rate and condition. Because environmental conditions can affect parental behavior and parents may mitigate environmental effects through altered behavior, we also used structural equation modeling (piecewiseSEM; Lefcheck, 2016; Lefcheck et al., 2020) to determine the direct and indirect effects of the environment and parental care on offspring. This multilevel approach allowed us to determine whether parents alter behavior in response to environmental conditions and whether these behaviors compensate for environmental influences on offspring development. Our work provides insight into how environmental conditions and parental care can interact to impact development. While we predicted that nest parasites and low arthropod availability would be directly associated with decreased nestling growth rate and hematocrit, we predicted that parents would alter behavior in response to environmental conditions and ameliorate the direct effects of the environment on offspring.

## 2 MATERIALS AND METHODS

We monitored 76 nest boxes in 2018 at three field sites and 97 nest boxes in 2019 at four field sites in Athens County, Ohio, USA, during the breeding season. Field sites were on private (two sites) and public land (two sites), ranged in size from 2 to 82 hectares (including the Ohio University Land Lab [39°19′N, 82°07′W]), and habitat included wetland, open field, and woods with small openings and varied in elevation (approximately 195–290 m). Nest boxes were constructed of wood and were approximately 15.5 cm wide by 30.5 cm tall. Some adults were color‐banded for individual identification if captured in mist nets as part of a broader study; however, we did not trap adult birds at the nests to avoid influencing nest box choice.

Because nestlings had to survive until at least day 6 for us to obtain growth rate and hematocrit, we included 69 Eastern Bluebird nests in this study. We monitored nests every one to three days from late April to the first week of August. During the nestling stage, we video‐recorded nests every other day between 0600 and 1200 for 90 min with Canon Vixia HF (R500 or R800) video cameras mounted on a tripod placed ~10 m from the nest to avoid altering parental behavior. For each day, we had video data, nestling age was recorded, and data were summed to calculate the total time adults spent at the nest (after the parent's head entered the box) divided by the amount of time the nest was observed (nest attentiveness = % time at the nest) and the total number of trips parents made to the nest divided by the time the nest was observed (provisioning rate). For consistency and to avoid missing data, we included video data of nests until nestlings were 6 or 7 days old in analyses. To determine incubation and brooding temperatures, we inserted Thermochron iButtons (model DS1921G‐F5; Embedded Data Systems, accuracy ±1°C) placed flush with the bottom of the nest cup after incubation began to record nest temperature every four minutes until we banded nestlings (6–12 days old), and every 10 min postbanding (to avoid disturbance at the nest and prevent early fledging) until the nest fledged or failed.

When foraging, Eastern Bluebirds predominantly use a perch within 0.5–15 m of the ground to search for prey items, then sally to capture prey from a substrate, usually the ground or vegetation on the ground, but also engage in flycatching and gleaning (Goldman, 1975; Gowaty & Plissner, 2020). Malaise traps can effectively sample food availability for insectivores when used in appropriate microhabitats (Wolda, 1990), and sample flying insects including adult Lepidoptera, Hymenoptera, and Diptera but are less effective at capturing Coleoptera and other ground arthropods (Cooper & Whitmore, 1990). When parents were feeding nestlings, we placed malaise traps within the foraging territory of parent birds for 48 h to quantify arthropod biomass available for feeding. Diptera made up the most individuals captured in our sampling (64.4%), followed by Lepidoptera (4.53%), Coleoptera (2.94%), Orthoptera (0.63%), and spiders (0.36%). While larval Lepidopterans are not usually sampled in malaise traps, the abundance of adult Lepidoptera is positively correlated with caterpillar abundance (Stange et al., 2011). We recorded the dry mass of each sample, then stored arthropods in 70% ethanol for later processing. We measured (length and width in mm) and identified arthropods to order. We estimated order Shannon diversity and Pileous evenness (Legendre & Legendre, 1998) in vegan (Oksanen et al., 2019). We assigned arthropods to one of four size classes by length: 1–10, 10–20, 20–30, and >30 mm. We estimated the volume of arthropods by size class by multiplying their width by 0.5π^2^*l, where *l* is the length of the insect (Blondel et al., 1991).

When nestlings were between 6 and 12 days old, we measured wing length, weighed nestlings (g), and collected a blood sample (<1% of nestling mass) in 75‐µl capillary tubes. We centrifuged blood samples to estimate hematocrit using a microhematocrit capillary tube reader. We searched for ectoparasites (blow flies, lice, and mites) on nestlings and in the nest during the nestling period, and we searched nests after fledging for lice, mites, and blow fly larvae and pupae. During the 2019 breeding season, we measured the tarsus (mm) of each nestling three times before nestling day 10. We calculated nestling growth rate (K) for tarsus following Sofaer et al. (2013) by defining the logistic function with nestling age as a covariate, then modeled nestling as a random effect in the nonlinear mixed‐effects model.

### 2.1 Statistical methods

Because of the large number of variables we collected that might explain variation in offspring development, we selected variables to include in candidate sets of models based on a priori hypotheses and limitations due to missing data. Nestling growth rate was positively correlated with hematocrit (β = 0.2559 ± 0.0934, *df* = 114, *t* = 2.74, *p* = .007), but because we only measured growth rate in 2019, we used growth rate and hematocrit as response variables in separate models. Similar to previous work (reviewed in Gowaty & Plissner, 2020), our data suggested similar provisioning rates of males (0.082 ± 0.001 trips per minute) and females (0.078 ± 0.001 trips per minute) so we included total provisioning rate. Because adult bluebirds predominantly feed small prey items to nestlings, we included the volume of small arthropods (<10 mm) instead of larger size classes. Food deserts (areas with low food availability) are associated with adverse health outcomes (reviewed in Gundersen & Ziliak, 2015; Walker et al., 2010), and arthropod availability is positively correlated with fecundity in insectivorous birds (Nagy & Holmes, 2004, 2005); therefore, we also included arthropod biomass (g). We used the presence or absence of blow flies in models instead of lice and mites because the latter two taxa may feed on skin or feathers instead of taking bloodmeals. Initial analyses also indicated that brood size was related to nest attendance and provisioning; however, brood size was not related to growth rate (β = −0.0225 ± 0.1523, *df* = 45, *t* = −0.15, *p* = .88) so we did not include brood size as a covariate in models of growth rate. We also used brood size as a covariate in all models of hematocrit because brood size and hematocrit were positively correlated (β = −0.2131 ± 0.1003, *df* = 60, *t* = −2.12, *p* = .04).

All analyses were conducted in R (ver. 4.0.3, R Core Team, 2020). We standardized all continuous predictor variables so our parameter estimates reflect effect size (Nieminen et al., 2013), and we report mean ± SE throughout. Because of missing data and to avoid overfitting, we developed several candidate sets of models (Tables S1–S4) and used an information‐theoretic approach to select the most supported models (Burnham & Anderson, 2002). We used mixed‐effects models in package nlme (Pinheiro et al., 2021) with nest ID as a random variable as a multivariate approach to repeated measures (Zuur et al., 2009) because estimates of provisioning rate, nest attendance, and nest temperatures were collected on multiple days at each nest. To find the most supported models, we first determined whether growth rate (Table S1) and hematocrit (Table S2) were associated with parental care (total provisioning rate [trips per minute] and nest attendance [proportion of time at the nest]) and environmental characteristics (blow fly presence, biomass availability, and volume of small arthropods). We developed another candidate set of models with growth rate and hematocrit as response variables with incubation and brooding temperatures (Tables S3 and S4). We did not run variables that were highly correlated (*r* > .7) in the same model, and we checked models for multicollinearity using the variance inflation factor (vif) in the car package (Fox et al., 2020). If vif indicated multicollinearity (>2), we reran models without that combination of variables (and thus do not include the models in our model selections tables). We fitted models using maximum likelihood, then used AICc and AICc weights in package MuMIn (Barton, 2019) to determine the most supported model for each candidate set. We then ran the AICc‐selected models from each candidate set with restricted maximum‐likelihood estimation to make inferences (Zuur et al., 2009).

While more traditional general linear mixed‐effects models (above) provide insight into the factors that explain variation in the response variable, we also wanted to understand the interplay between the environment and parental care on offspring and determine whether parents could compensate for poor environmental conditions. We used piecewiseSEM (Lefcheck et al., 2020) because these models can include a random effect. We used nest ID as a random variable to determine the direct and indirect effects of the natal environment and parental care on nestling hematocrit and growth rate in separate models. We tested the following predictions related to growth rate:

- Nestling growth rate was directly affected by environmental conditions (arthropod biomass, volume of small arthropods, and blow fly parasitism) and parental care (provisioning rate, nest attentiveness and nest temperature). We included biomass as a direct effect to nestling growth rate because under poor resource conditions (like a food desert), parents could have insufficient food to provide to nestlings because of the environment in which they live.
- Provisioning rate was affected by environmental conditions (arthropod biomass, volume of small arthropods, and blow fly parasitism).
- Nest attendance was affected by environmental conditions (arthropod biomass, volume of small arthropods, and blow fly parasitism).
- Brooding temperature was affected by environmental conditions (arthropod biomass, volume of small arthropods, and blow fly parasitism).

We also modeled correlations between provisioning rate and brooding temperature, brooding temperature and brood size, nest attendance and provisioning rate, provisioning rate and brood size, volume of small arthropods, and arthropod biomass.

We tested the following predictions related to hematocrit:

- Nestling hematocrit was directly affected by environmental conditions (arthropod biomass, volume of small arthropods, and blow fly parasitism) and parental care (provisioning rate, nest attentiveness, and nest temperature).
- Provisioning rate was affected by environmental conditions (arthropod biomass, volume of small arthropods, and blow fly parasitism).
- Nest attendance was affected by environmental conditions (arthropod biomass, volume of small arthropods, and blow fly parasitism).
- Brooding temperature was affected by environmental conditions (arthropod biomass, volume of small arthropods, and blow fly parasitism).

We also modeled correlations between provisioning rate and brooding temperature, brooding temperature and brood size, nest attendance and provisioning rate, provisioning rate and brood size, volume of small arthropods and arthropod biomass, and brooding temperature and nest attendance. For both response variables, we determined which correlations should be included using the tests of directed separation and compared models with AIC and evaluated model fit with Fisher's C to find the most supported model with *df* ≥ 1. Data and the code to replicate the structural equation models are available on a GitHub repository at https://github.com/ecologykelly/SemEcoTutorial.

## 3 RESULTS

### 3.1 Influence of environmental conditions on nestling growth and condition

**FIGURE 1:**
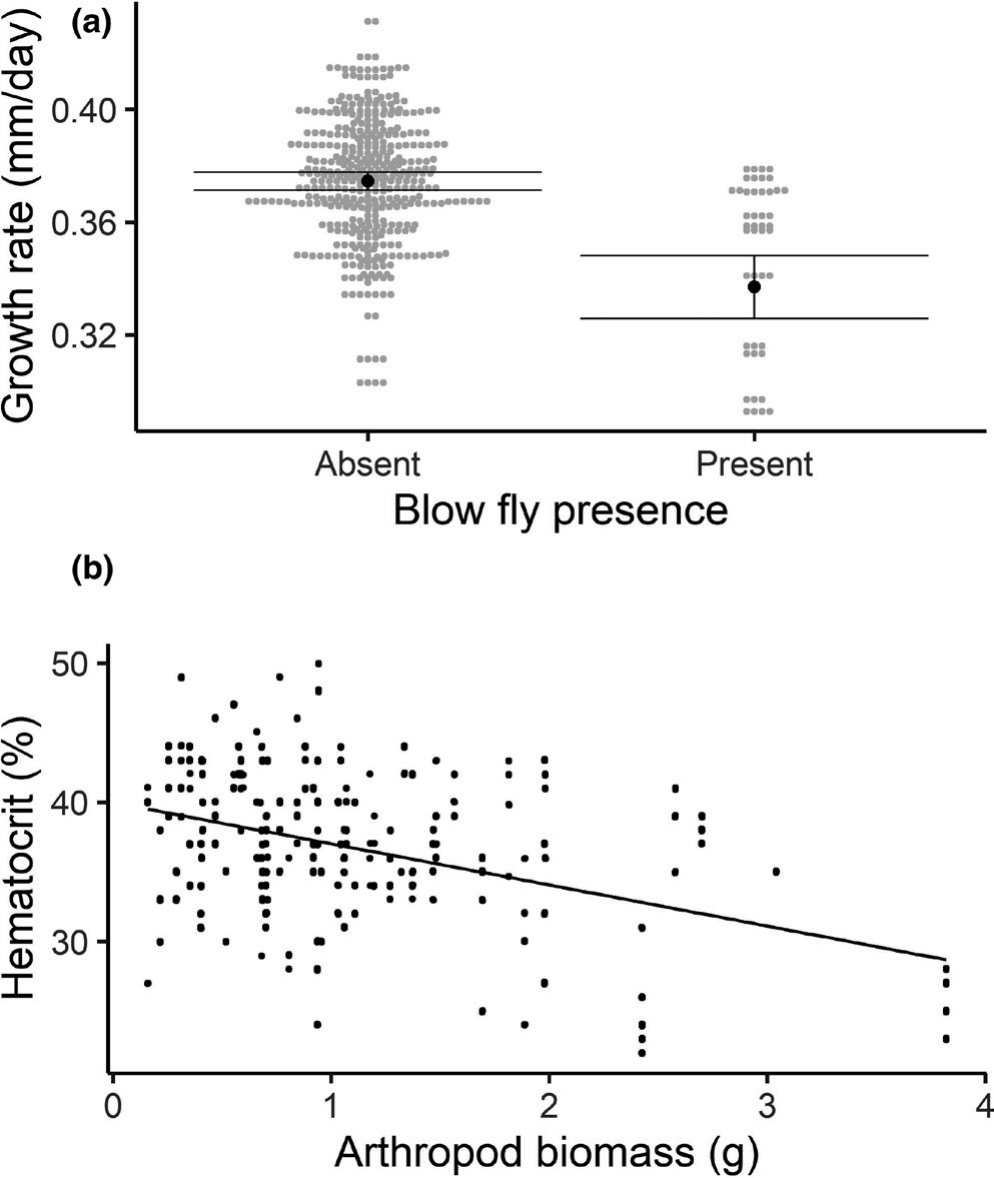
(a) Nestling hematocrit in relation to arthropod biomass (g). Fitted lines represent the predicted relationship from the model of hematocrit with biomass availability and brood size as predictor variables and nest ID as a random effect plotted over the raw data. (b) The growth rate (mean ± SE) of nestlings in nests without blow flies (absent; 0.34 mm/day ± 0.009 mm/day) or with blow fly parasitism (present; 0.38 ± 0.002 mm/day; *t* = −3.64, *p* = .003). Marginal means and SE are plotted from the model of growth rate with blow fly presence and biomass availability as predictor variables and nest ID as a random effect. Data points are plotted from the raw data

We included 38 nests (444 observations from 146 nestlings) in our analysis of the impact of environmental conditions and parental care (Table 1) on growth rate. Environmental variables in the AICc‐selected model included biomass availability, blow fly presence, and volume of small arthropods (Table S1). We found that growth rate was not related to the volume of small arthropods (β = 0.2068 ± 0.1232, *t* = 1.67, *df* = 404, *p* = .09) nor biomass availability (β = 0.1754 ± 0.0924, *t* = 1.89, *df* = 404, *p* = .06); however, nestling growth rate was lower in nests parasitized by blow flies (0.34 ± 0.009 mm/day) than nestlings that were in nests without blow flies (0.38 ± 0.002 mm/day; β = −1.6899 ± 0.4641, *t* = −3.64, *df* = 36, *p* = .003; Figure 1a).

**TABLE 1:** Descriptive statistics (mean ± SE) and range of variables included in AICc‐selected models

Variable	Mean ± SE	Range
Growth rate	0.37 ± 0.002 mm/day	0.29–0.43 mm/day
Hematocrit	37.06% ± 0.35%	22%–50%
Arthropod biomass	1.28 g ± 0.10 g	0.16–3.82 g
Volume of small arthropods	838.76 ± 74.71 mm^3^	58.88–2812.26 mm^3^
Incubation temperature	30.31°C ± 0.41°C	23.99–36.91°C
Provisioning rate	0.164 ± 0.0540 trips/min	0.0–0.43 trips/min
Nest attendance	32.0 ± 13.0% time at nest	0.0%–87% time at nest
Blow fly parasitism	8.70% parasitized	NA

Note

Blow fly parasitism is percentage of nests parasitized.

We included 49 nests (674 observations from 236 nestlings) in our analysis of the impact of environmental conditions and parental care on hematocrit. Environmental variables in the top model included biomass availability, blow fly presence, and brood size (Table S2). There was no difference in hematocrit between nests with (32.81 ± 0.72%) and without (37.15 ± 0.21%; β = −0.5314 ± 0.2784, *t* = −1.91, *df* = 46, *p* = .06) blow flies, and there was no relationship between hematocrit and brood size (β = −0.1228 ± 0.1131, *t* = −1.09, *df* = 46, *p* = .28), but increased biomass availability was associated with lower hematocrit (β = −0.1512 ± 0.0693, *t* = −2.18, *df* = 404, *p* = .03; Figure 1b).

### 3.2 Influence of nest temperature on nestling growth and condition

We included 40 nests (484 observations from 144 nestlings) in our analysis of the impact of nest temperature on growth rate. The top model only included average incubation temperature (Table S3), and there was no relationship between growth rate and incubation temperature (β = −0.0724 ± 0.1886, *t* = −0.38, *df* = 38, *p* = .70).

We included 50 nests (642 observations from 178 nestlings) in our analysis of the impact of nest temperature on hematocrit. The top model included average incubation temperature and brood size (Table S4). There was no relationship between hematocrit and incubation temperature (β = −0.1314 ± 0.1118, *t* = −1.17, *df* = 47, *p* = .24) nor brood size (β = −0.1218 ± 0.1170, *t* = −1.04, *df* = 47, *p* = .30).

### 3.3 Direct and indirect effects on growth rate (piecewiseSEM)

**FIGURE 2:**
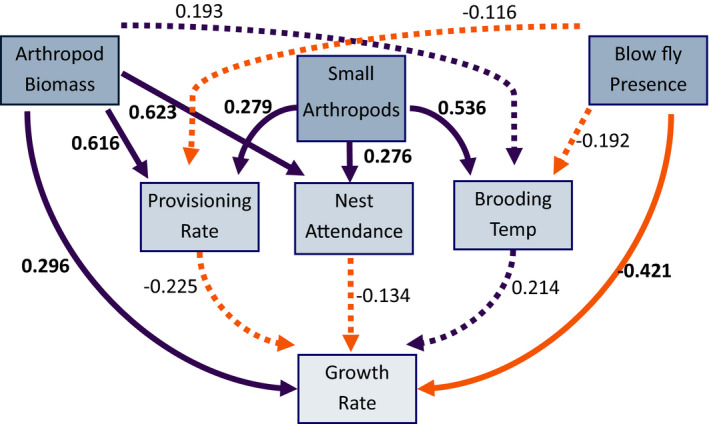
Effects of environmental factors (blow fly presence, volume small arthropods [mm^3^], and arthropod availability [g]) and parental care (provisioning rate, nest attendance, and nest temperature) on growth rate from our piecewiseSEM with nest ID as a random effect. Boxes represent measured variables. Arrows represent unidirectional relationships among variables. Orange arrows denote negative relationships, purple arrows denote positive relationships, solid lines indicate significant relationships (*p* < .05), and dashed lines indicate non‐significant paths. Standardized path coefficients (effect size) are indicated and are in bold if *p* < .05

The selected piecewiseSEM (Table S5) explained 67% of variation in growth (AIC = 54.84, Fisher's C = 4.84, *df* = 6, *p* = .56, 149 nestlings from 40 nests). Territories with more available arthropod biomass had nestlings with higher growth rates (standardized estimate: β = 0.2955, *p* = .04; Table 2, Figure 2). Nestlings in nests parasitized by blow flies had slower growth rates (β = −0.4206, *p* = .004). Growth rate was not directly influenced by any parental care behaviors that we examined including nest attendance (β = −0.1343, *p* = .29), provisioning rate (β = −0.2249, *p* = .1223), or brooding temperature (β = 0.2145, *p* = .12). While arthropod biomass was not related to brooding temperature (β = 0.1932, *p* = .34), parents on territories with greater biomass had higher provisioning rates (β = 0.5162, *p* = .003) and nest attendance (β = 0.6225, *p* = .0002). The volume of small arthropods was positively associated with nest attendance (β = 0.2760, *p* = .003), brooding temperature (β = 0.5361, *p* = .003), and provisioning rate (β = 0.2791, *p* = .01). There was no relationship between blow fly presence and nest temperature (β = −0.1921, *p* = .34) or provisioning (β = −0.1160, *p* = .50).

**TABLE 2:** PiecewiseSEM model of factors influencing offspring growth rate (growth), provisioning rate (provisioning), nest attendance (attendance), and brooding temperature (temp) with nest ID as a random effect and modeled correlations (~~)

Response	Predictor	Estimate	SE	*df*	*p*	Effect size
Growth rate	**Blow flies**	−0.0374	0.0122	34	.0041	−0.4206
	**Arthropod mass**	0.008	0.0038	34	.0409	0.2955
	Provisioning	−0.1068	0.0674	34	.1223	−0.2249
	Nest attendance	−0.0296	0.0273	34	.2865	−0.1343
	Temp	0.0016	0.001	34	.1155	0.2145
Provisioning	**Small arthropods**	0	0	36	.0103	0.2791
	**Arthropod mass**	0.0296	0.0092	36	.0028	0.5162
	Blow flies	−0.0217	0.032	36	.5018	−0.116
Nest attendance	**Small arthropods**	0.0001	0	37	.0033	0.276
	**Arthropod mass**	0.077	0.0189	37	.0002	0.6225
Temp	**Small arthropods**	0.0033	0.001	36	.0026	0.5361
	Arthropod mass	0.719	0.7433	36	.3398	0.1932
	Blow flies	−2.3353	2.4386	36	.3446	−0.1921
~~Provisioning	~~Temp	0.1027		149	.1071	0.1027
~~Temp	~~Brood	−0.033		149	.3453	−0.033
~~Temp	~~Provisioning	−0.009		149	.4569	−0.009
~~Provisioning	~~Brood	−0.0686		149	.2038	−0.0686
~~Small arthropods	~~Arthropod mass	0.0261		147	.752	0.0261

Note

Predictor variables including arthropod biomass (arthropod mass), volume of arthropods 1–10 mm (small arthropods), and blow fly presence (blow fly) are listed with parameter estimates, standard error (SE), degrees of freedom (*df*), *p*‐value, and the standardized coefficient (effect size). Significant terms (*p* < .05) are highlighted in bold.

### 3.4 Direct and indirect effects on hematocrit (piecewiseSEM)

**FIGURE 3:**
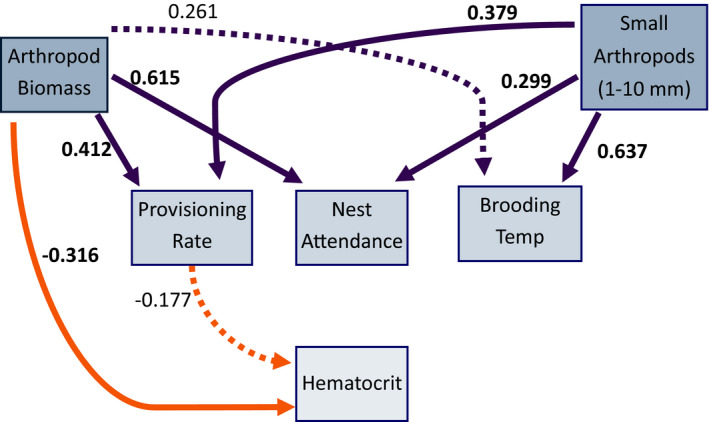
Effects of environmental factors (volume of small arthropods [mm^3^] and arthropod availability [g]) and parental care (provisioning rate, nest attendance, and nest temperature) on hematocrit from our piecewiseSEM. Boxes represent measured variables. Arrows represent unidirectional relationships among variables. Orange arrows denote negative relationships, purple arrows positive relationships, solid lines indicate significant relationships (*p* < .05), and dashed lines indicate non‐significant paths. Standardized path coefficients (effect size) are indicated and are in bold if *p* < .05

The piecewiseSEM (Table S6) explained 52% of variation in hematocrit (AIC = 44.88, Fisher's C = 1.77, *df* = 6, *p* = .56, 179 nestlings from 49 nests). Arthropod biomass availability was negatively associated with hematocrit (β = −0.3162, *p* = .005), but positively associated with provisioning rate (β = 0.4118, *p* = .005) and nest attendance (β = 0.6152, *p* < .0001; Table 3, Figure 3). There was no relationship between biomass availability and brooding temperature (β = 0.2605, *p* = .13). The volume of small arthropods was positively associated with nest attendance (β = 0.2993, *p* = .003), brooding temperature (β = 0.6365, *p* = .0002), and provisioning rate (β = 0.3789, *p* = .001). Hematocrit was not influenced by provisioning rate (β = −0.1766, *p* = .10).

**TABLE 3:** PiecewiseSEM model of factors influencing offspring hematocrit (hem), provisioning rate (provisioning), nest attendance (attendance), and brooding temperature (temp) with nest ID as a random effect and modeled correlations (~~)

Response	Predictor	Estimate	SE	*df*	*p*	Effect size
Hem	**Arthropod mass**	−2.2084	0.7544	46	.0053	−0.3162
	Provisioning	−18.0485	10.9027	46	.1046	−0.1766
Provisioning	**Small arthropods**	0	0	46	.0014	0.3789
	**Arthropod mass**	0.0281	0.0096	46	.0053	0.4118
Attendance	**Small arthropods**	0.0001	0	46	.0033	0.2993
	**Arthropod mass**	0.0941	0.0199	46	0	0.6152
Temp	**Small arthropods**	0.0036	0.0009	46	.0002	0.6365
	Arthropod mass	1.2331	0.7921	46	.1264	0.2605
~~Provisioning	**~~Temp**	0.2343	–	179	.0008	0.2343
~~Temp	**~~Brood**	−0.2442	–	179	.0005	−0.2442
~~Attendance	**~~Provisioning**	0.1772	–	179	.009	0.1772
~~Provisioning	**~~Brood**	−0.1962	–	179	.0043	−0.1962
~~Small arthropods	~~Arthropod mass	0.0191	–	177	.7992	−0.0191
~~Temp	**~~Attendance**	−0.1243	–	179	.0491	−0.1243

Note

Predictor variables including arthropod biomass (arthropod mass), volume of arthropods 1–10 mm (small arthropods), and blow fly presence (blow fly) are listed with parameter estimates, standard error (SE), degrees of freedom (*df*), *p*‐value, and the standardized coefficient (effect size). Significant terms (*p* < .05) are highlighted in bold.

## 4 DISCUSSION

Environmental conditions directly affected offspring growth and condition. Parents did not alter behavior in response to parasite presence, resulting in reduced offspring growth and hematocrit. While parental care behavior increased under favorable conditions (increased food availability), parents did not compensate for low food availability and thus had offspring with lower growth rate and hematocrit. While previous research has explored the possibility for behavioral alteration in response to environmental variation (Sih, 2013) and behavioral rescue (Fey et al., 2019), our work highlights the importance of environmental conditions to offspring development and suggests parents may be unable to sufficiently alter behavior to ameliorate challenging environments.

### 4.1 Environmental impacts on development

In nests parasitized by blow flies, nestlings had decreased growth rates compared with nests without blow flies; however, blow fly parasitism did not influence hematocrit. Parasite presence influences trade‐offs between growth and condition of nestling birds (O’Brien & Dawson, 2008). If current survival is prioritized, nestlings can allocate available resources to maintain growth (Kersten & Brenninkmeijer, 1995). Nestlings in our system could be allocating resources to maintain hematocrit, which is related to increased oxygen‐carrying capacity, metabolic rate, and flight performance (Petit & Vezina, 2014; Yap et al., 2018). In addition, developmental responses can be diverse: In nests parasitized by louse flies, nestling Barn Swallows had faster feather growth but poorer condition than those without parasites (Saino et al., 1998). In contrast, fleas reduced feather growth but had no impact on body condition (Tripet & Richner, 1997).

Nestling bluebirds had higher growth rates but lower hematocrit on territories with greater food availability. Environments with increased prey were related to increased daily mass gain in Gambel's White‐crowned Sparrow (*Zonotrichia leucophrys gambelii*) and Lapland Longspur (*Calcarius lapponicus*; Pérez et al., 2016). Our finding that increased arthropod availability had a negative impact on hematocrit is contrary to studies that have found improved condition related to increased food availability (Bradbury et al., 2003); however, we cannot rule out the possibility that higher hematocrit levels were related to dehydration (Vleck & Priedkalns, 1985). In addition, we did not estimate food quality, but food quality may have impacted condition in the birds we studied. Low‐quality food is associated with decreased measures of avian condition, such as immune function (Cummings et al., 2020) and body mass (Wright et al., 1998). Similarly, when European Starlings brought larger portions of low‐quality food, nestling growth rate was slower than when parents provided more digestible food (Wright et al., 1998).

### 4.2 Environmental impacts on parental care

Parents on territories with more food biomass increased provisioning rate and nest attendance. When more food is available, parents are able to locate prey more easily (Tremblay et al., 2005) resulting in increased provisioning rates, nest attendance, or self‐care behaviors (Low et al., 2012; Moreno et al., 1999). For example, Eurasian reed warblers (*Acrocephalus scirpaceus*) and Karoo Prinia (*Prinia maculosa*) experimentally supplemented with food were able to increase time at the nest compared with controls (Chalfoun & Martin, 2007; Vafidis et al., 2018). In contrast, environmental conditions can limit provisioning (Williams & DeLeon, 2020). Search time increases with decreasing prey biomass (Naef‐Daenzer & Keller, 1999; Naef‐Daenzer et al., 2001) and can force trade‐offs among the adult's survival and parental behaviors. When resource availability is high, the costs of providing more food may be minimal compared with the benefit to nestlings. However, the additional costs of increased provisioning under low food availability may not result in a proportional benefit to offspring.

Higher abundance of small arthropods in a territory led to increased nest attendance, higher average brooding temperatures, and greater provisioning rates. Great Tit (*Parus major*) parents had decreased daily energy expenditure when there was greater availability of preferred prey (Tinbergen & Dietz, 1994), and parents supplemented with appropriate prey had higher nest attendance and greater incubation constancy (Vafidis et al., 2018). In contrast, birds faced with lower availability of prey items spent a smaller proportion of time on the nest (Caldow & Furness, 2000).

Parasitism by blow flies did not alter provisioning rate, nest attendance, or brooding temperature. While altered parental behavior due to parasitism is common (Bouslama et al., 2002), not all parents are able to compensate for parasitism with increased provisioning (Cantarero et al., 2013; Walker & Rotherham, 2011; Williams & DeLeon, 2020), and some may prioritize self‐maintenance rather than increasing offspring care (Wegmann et al., 2015). Parents increase provisioning in response to nestling cues, such as begging, which can be reduced in offspring weakened by blood‐feeding parasites (Moreno‐Rueda et al., 2016). Therefore, parents may be limited in how they can respond to parasitism as cues needed to alter behavior may not accurately reflect the nutritional state of parasitized nestlings.

### 4.3 Impact of parental care on development

While we predicted that parents would adjust behavior to mitigate impacts of environmental conditions on offspring, we found that parental behaviors were not able to overcome the environmental influences on growth rate or hematocrit. Parents increased parental care in response to favorable environmental conditions; however, nest attendance, provisioning rate, and nest temperature had no direct relationship to offspring growth or hematocrit. Bluebird parents had decreased provisioning when prey availability was low, suggesting that parents experienced trade‐offs with other behaviors. When challenged, parents may not work harder due to trade‐offs among parental care behaviors, self‐maintenance, and future reproduction (Hill, 2003; Williams, 1966), and even when parents lost body mass while providing for chicks in low‐resource areas, chicks had higher starvation rates (Numata et al., 2004). Parents may also be limited in how they can compensate for different environmental conditions. For example, the small tree finch (*Camarhynchus parvulus*) did not increase provisioning in response to parasitism and provisioned less under rainy conditions but may have compensated for poor food quality with increased provisioning rates (Heyer et al., 2021). Parasitism also can alter the activity budget: Blow fly parasitism was associated with increased nest sanitation behaviors but reduced overall nest attendance; however, females were not observed removing larvae (Hurtrez‐Boussès et al., 2000).

We did not find any relationship between nest attendance or brooding temperature and offspring development. Nest attendance by parents can shelter offspring from weather including cold temperature or precipitation (Laux et al., 2016), may reduce nest predation (Conway & Martin, 2000; Fontaine & Martin, 2006), and can influence nest temperature (Williams et al., 2020). Temperatures outside of an appropriate range lead to slower growth and dehydration (Salaberria et al., 2014). Poor nest attendance and nest temperatures lead to early death of offspring (Webb, 1987), and offspring in our study that failed to hatch or died before growth rate and hematocrit could be measured were not included in our analyses. Thus, we may not have found a relationship between nest attendance or temperature and offspring development because parents that were able to raise chicks to 6–12 days may have had parental care behaviors in the range sufficient for offspring survival.

## 5 CONCLUSION

It is often predicted that behavioral flexibility will ameliorate the effects of the environment on fitness measures (Fey et al., 2019; Hurtrez‐Boussès et al., 2000); however, our work suggests that while parental care behavior is modified in relation to environmental factors, the changes in behavior do not completely compensate for the effects of the environment on offspring growth and condition. Our results also highlight the importance of the environment and ecosystem function on demography. As worldwide insect populations decline, insect biomass could be a critical factor in maintaining bird populations. Human‐altered habitats can present novel situations that parents are unprepared or unable to adapt to (Sih, 2013), and the plasticity parents have in their behaviors may not be enough to compensate for poor environmental conditions (Wong & Candolin, 2015). Understanding how parents respond to environmental variation may help predict whether the impacts of altered habitats on offspring can be mitigated.

## CONFLICT OF INTEREST

None declared.

## AUTHOR CONTRIBUTION

**Madeline Cassidy Sudnick:** Conceptualization (equal); data curation (equal); formal analysis (equal); funding acquisition (equal); investigation (equal); methodology (equal); project administration (equal); validation (equal); visualization (equal); writing–original draft (equal); writing–review and editing (equal). **Bekka S. Brodie:** Funding acquisition (supporting); methodology (supporting); resources (supporting); writing–review and editing (supporting). **Kelly A. Williams:** Conceptualization (lead); data curation (equal); formal analysis (equal); funding acquisition (lead); investigation (equal); methodology (equal); project administration (lead); resources (equal); supervision (lead); validation (equal); visualization (equal); writing–original draft (equal); writing–review and editing (equal).

## Supporting information

Supplementary Material

Supplementary Material

## Data Availability

All data are archived in Dryad https://doi.org/10.5061/dryad.63xsj3v3c. Data and code for the structural equation models are available at https://github.com/ecologykelly/SemEcoTutorial

## References

[ece38207-bib-0001] Ardia, D. R. (2013). The effects of nestbox thermal environment on fledging success and haematocrit in Tree Swallows. Avian Biology Research, 6(2), 99–103. 10.3184/175815513X13609528031394

[ece38207-bib-0002] Ardia, D. R. , Pérez, J. H. , Chad, E. K. , Voss, M. A. , & Clotfelter, E. D. (2009). Temperature and life history: Experimental heating leads female tree swallows to modulate egg temperature and incubation behaviour. Journal of Animal Ecology, 78(1), 4–13. 10.1111/j.1365-2656.2008.01453.x 18637971

[ece38207-bib-0003] Barton, K. (2019). MuMIn: Multi‐Model Inference (R package version 1.43.6) [Computer software]. https://CRAN.R‐project.org/package=MuMIn

[ece38207-bib-0004] Beltrán, I. , Loiseleur, R. , Durand, V. , & Whiting, M. J. (2020). Effects of early thermal environment on the behavior and learning of a lizard with bimodal reproduction. Behavioral Ecology and Sociobiology, 74(6), 73. 10.1007/s00265-020-02849-6 32951106

[ece38207-bib-0005] Blondel, J. , Dervieux, A. , Maistre, M. , & Perret, P. (1991). Feeding ecology and life history variation of the Blue Tit in Mediterranean deciduous and sclerophyllous habitats. Oecologia, 88(1), 9–14. 10.1007/BF00328397 28312725

[ece38207-bib-0006] Bouslama, Z. , Lambrechts, M. M. , Ziane, N. , Djenidi, R. , & Chabi, Y. (2002). The effect of nest ectoparasites on parental provisioning in a north‐African population of the Blue Tit *Parus caeruleus* . Ibis, 144(2), E73–E78. 10.1046/j.1474-919X.2002.00070_5.x

[ece38207-bib-0007] Bowen, W. D. , den Heyer, C. E. , McMillan, J. I. , & Iverson, S. J. (2015). Offspring size at weaning affects survival to recruitment and reproductive performance of primiparous gray seals. Ecology and Evolution, 5(7), 1412–1424. 10.1002/ece3.1450 25897381 PMC4395171

[ece38207-bib-0009] Bradbury, R. B. , Wilson, J. D. , Moorcroft, D. , Morris, A. J. , & Perkins, A. J. (2003). Habitat and weather are weak correlates of nestling condition and growth rates of four UK farmland passerines. Ibis, 145(2), 295–306. 10.1046/j.1474-919X.2003.00142.x

[ece38207-bib-0010] Brinkerhoff, R. J. , Haddad, N. M. , & Orrock, J. L. (2005). Corridors and olfactory predator cues affect small mammal behavior. Journal of Mammalogy, 86(4), 662–669.

[ece38207-bib-0011] Brommer, J. E. , Pitala, N. , Siitari, H. , Kluen, E. , & Gustafsson, L. (2011). Body size and immune defense of nestling Blue Tits (*Cyanistes caeruleus*) in response to manipulation of ectoparasites and food supply. The Auk, 128(3), 556–563. 10.1525/auk.2011.10284

[ece38207-bib-0012] Brown, C. R. , & Brown, M. B. (1986). Ectoparasitism as a cost of coloniality in Cliff Swallows (*Hirundo Pyrrhonota*). Ecology, 67(5), 1206–1218. 10.2307/1938676

[ece38207-bib-0013] Burnham, B. A. , & Anderson, D. R. (2002). Model selection and multimodel inference: A practical information‐theoretic approach (2nd ed.). Springer Science and Business Media LLC.

[ece38207-bib-0014] Caldow, R. W. G. , & Furness, R. W. (2000). The effect of food availability on the foraging behaviour of breeding Great Skuas *Catharacta skua* and Arctic Skuas *Stercorarius parasiticus* . Journal of Avian Biology, 31(3), 367–375. 10.1034/j.1600-048X.2000.310313.x

[ece38207-bib-0015] Cantarero, A. , López‐Arrabé, J. , Redondo, A. J. , & Moreno, J. (2013). Behavioural responses to ectoparasites in pied flycatchers *Ficedula hypoleuca*: An experimental study. Journal of Avian Biology, 44(6), 591–599. 10.1111/j.1600-048X.2013.00134.x

[ece38207-bib-0016] Chalfoun, A. D. , & Martin, T. E. (2007). Latitudinal variation in avian incubation attentiveness and a test of the food limitation hypothesis. Animal Behaviour, 73(4), 579–585. 10.1016/j.anbehav.2006.09.010

[ece38207-bib-0017] Conway, C. J. , & Martin, T. E. (2000). Evolution of passerine incubation behavior: Influence of food, temperature, and nest predation. Evolution, 54(2), 670–685. 10.1111/j.0014-3820.2000.tb00068.x 10937242

[ece38207-bib-0098] Cooper, R. , & Whitmore, R. C. (1990). Arthropod sampling methods in Ornithology. Studies in Avian Biology, 13, 29–37.

[ece38207-bib-0018] Cummings, C. R. , Hernandez, S. M. , Murray, M. , Ellison, T. , Adams, H. C. , Cooper, R. E. , Curry, S. , & Navara, K. J. (2020). Effects of an anthropogenic diet on indicators of physiological challenge and immunity of white ibis nestlings raised in captivity. Ecology and Evolution, 10(15), 8416–8428. 10.1002/ece3.6548 32788990 PMC7417218

[ece38207-bib-0019] Deeming, D. C. , & Reynolds, S. J. (2015). Nests, eggs, and incubation: New ideas about Avian reproduction. Oxford University Press.

[ece38207-bib-0020] Eggert, A.‐K. , Reinking, M. , & Müller, J. K. (1998). Parental care improves offspring survival and growth in burying beetles. Animal Behaviour, 55(1), 97–107. 10.1006/anbe.1997.0588 9480676

[ece38207-bib-0021] Emlen, S. T. , Wrege, P. H. , Demong, N. J. , & Hegner, R. E. (1991). Flexible growth rates in nestling White‐fronted Bee‐eaters: A possible adaptation to short‐term food shortage. The Condor, 93(3), 591–597. 10.2307/1368191

[ece38207-bib-0022] Fey, S. B. , Vasseur, D. A. , Alujević, K. , Kroeker, K. J. , Logan, M. L. , O’Connor, M. I. , Rudolf, V. H. W. , DeLong, J. P. , Peacor, S. , Selden, R. L. , Sih, A. , & Clusella‐Trullas, S. (2019). Opportunities for behavioral rescue under rapid environmental change. Global Change Biology, 25(9), 3110–3120. 10.1111/gcb.14712 31148329

[ece38207-bib-0023] Fontaine, J. J. , & Martin, T. E. (2006). Parent birds assess nest predation risk and adjust their reproductive strategies. Ecology Letters, 9(4), 428–434. 10.1111/j.1461-0248.2006.00892.x 16623728

[ece38207-bib-0024] Fox, J. , Weisberg, S. , Price, B. , Adler, D. , Bates, D. , Baud‐Bovy, G. , Bolker, B. , Ellison, S. , Firth, D. , Friendly, M. , Gorjanc, G. , Graves, S. , Heiberger, R. , Krivitsky, P. , Laboissiere, R. , Maechler, M. , Monette, G. , Murdoch, D. , Nilsson, H. , … R‐Core . (2020). car: Companion to Applied Regression (3.0‐10) [Computer software]. https://CRAN.R‐project.org/package=car

[ece38207-bib-0025] Goldman, P. (1975). Hunting behavior of Eastern Bluebirds. The Auk, 92(4), 798–801. 10.2307/4084793

[ece38207-bib-0026] Gowaty, P. A. , & Plissner, J. H. (2020). Eastern Bluebird (*Sialia sialis*), version 1.0. In A. F. Poole (Ed.), Birds of the World. Cornell Lab of Ornithology. 10.2173/bow.easblu.01

[ece38207-bib-0027] Grew, R. , Ratz, T. , Richardson, J. , & Smiseth, P. T. (2019). Parental care buffers against effects of ambient temperature on offspring performance in an insect. Behavioral Ecology, 30(5), 1443–1450. 10.1093/beheco/arz100

[ece38207-bib-0028] Gundersen, C. , & Ziliak, J. P. (2015). Food insecurity and health outcomes. Health Affairs, 34(11), 1830–1839. 10.1377/hlthaff.2015.0645 26526240

[ece38207-bib-0029] Hart, J. D. , Milsom, T. P. , Fisher, G. , Wilkins, V. , Moreby, S. J. , Murray, A. W. A. , & Robertson, P. A. (2006). The relationship between yellowhammer breeding performance, arthropod abundance and insecticide applications on arable farmland. Journal of Applied Ecology, 43(1), 81–91. 10.1111/j.1365-2664.2005.01103.x

[ece38207-bib-0030] Hepp, G. R. , Kennamer, R. A. , & Johnson, M. H. (2006). Maternal effects in Wood Ducks: Incubation temperature influences incubation period and neonate phenotype. Functional Ecology, 20(2), 308–314. 10.1111/j.1365-2435.2006.01108.x

[ece38207-bib-0031] Heyer, E. , Cimadom, A. , Wappl, C. , & Tebbich, S. (2021). Parental care in the Small Tree Finch *Camarhynchus parvulus* in relation to parasitism and environmental factors. Ibis, 163(1), 137–149. 10.1111/ibi.12845 33362293 PMC7754105

[ece38207-bib-0032] Hill, H. (2003). Adjustments in parental care by the European starling (*Sturnus vulgaris*): The effect of female condition. In Proceedings of The National Conference on Undergraduate Research (NCUR). University of Utah, Salt Lake City, Utah.

[ece38207-bib-0033] Hoi‐Leitner, M. , Romero‐Pujante, M. , Hoi, H. , & Pavlova, A. (2001). Food availability and immune capacity in serin (*Serinus serinus*) nestlings. Behavioral Ecology and Sociobiology, 49(5), 333–339. 10.1007/s002650000310

[ece38207-bib-0034] Hopkins, B. C. , DuRant, S. E. , Hepp, G. R. , & Hopkins, W. A. (2011). Incubation temperature influences locomotor performance in young wood ducks (*Aix sponsa*). Journal of Experimental Zoology Part A: Ecological Genetics and Physiology, 315A(5), 274–279. 10.1002/jez.673 21370488

[ece38207-bib-0035] Hurtrez‐Boussès, S. , Renaud, F. , Blondel, J. , Perret, P. , & Galan, M.‐J. (2000). Effects of ectoparasites of young on parents’ behaviour in a Mediterranean population of Blue Tits. Journal of Avian Biology, 31(2), 266–269. 10.1034/j.1600-048X.2000.310219.x

[ece38207-bib-0037] Kersten, M. , & Brenninkmeijer, A. (1995). Growth, fledging success and post‐fledging survival of juvenile Oystercatchers *Haematopus ostralegus* . Ibis, 137(3), 396–404. 10.1111/j.1474-919X.1995.tb08039.x

[ece38207-bib-0038] Klug, H. , & Bonsall, M. B. (2014). What are the benefits of parental care? The importance of parental effects on developmental rate. Ecology and Evolution, 4(12), 2330–2351. 10.1002/ece3.1083 25360271 PMC4203283

[ece38207-bib-0039] Krause, E. T. , Steinfartz, S. , & Caspers, B. A. (2011). Poor nutritional conditions during the early larval stage reduce risk‐taking activities of fire salamander larvae *(Salamandra salamandra*). Ethology, 117(5), 416–421. 10.1111/j.1439-0310.2011.01886.x

[ece38207-bib-0041] Laux, C. M. , Nordell, C. J. , Fisher, R. J. , Ng, J. W. , Wellicome, T. I. , & Bayne, E. M. (2016). Ferruginous Hawks *Buteo regalis* alter parental behaviours in response to approaching storms. Journal of Ornithology, 157(1), 355–362. 10.1007/s10336-015-1288-0

[ece38207-bib-0042] Lefcheck, J. S. (2016). piecewiseSEM: Piecewise structural equation modelling in r for ecology, evolution, and systematics. Methods in Ecology and Evolution, 7(5), 573–579. 10.1111/2041-210X.12512

[ece38207-bib-0043] Lefcheck, J. , Byrnes, J. , & Grace, J. (2020). piecewiseSEM: Piecewise Structural Equation Modeling (2.1.2) [Computer software]. https://CRAN.R‐project.org/package=piecewiseSEM

[ece38207-bib-0044] Legendre, P. , & Legendre, L. (1998). Numerical ecology (2nd English ed., p. 852). Elsevier.

[ece38207-bib-0045] Lindström, J. (1999). Early development and fitness in birds and mammals. Trends in Ecology & Evolution, 14(9), 343–348. 10.1016/S0169-5347(99)01639-0 10441307

[ece38207-bib-0046] Low, M. , Makan, T. , & Castro, I. (2012). Food availability and offspring demand influence sex‐specific patterns and repeatability of parental provisioning. Behavioral Ecology, 23(1), 25–34. 10.1093/beheco/arr145

[ece38207-bib-0047] McKinnon, L. , Picotin, M. , Bolduc, E. , Juillet, C. , & Bêty, J. (2012). Timing of breeding, peak food availability, and effects of mismatch on chick growth in birds nesting in the High Arctic. Canadian Journal of Zoology, 90(8), 961–971. 10.1139/z2012-064

[ece38207-bib-0048] Merino, S. , & Potti, J. (1995). Mites and blowflies decrease growth and survival in nestling Pied Flycatchers. Oikos, 73(1), 95–103. 10.2307/3545730

[ece38207-bib-0049] Moreno, J. , Merino, S. , Potti, J. , de León, A. , & Rodríguez, R. (1999). Maternal energy expenditure does not change with flight costs or food availability in the pied flycatcher (*Ficedula hypoleuca*): Costs and benefits for nestlings. Behavioral Ecology and Sociobiology, 46(4), 244–251. 10.1007/s002650050616

[ece38207-bib-0050] Moreno‐Rueda, G. , Redondo, T. , Ochoa, D. , Camacho, C. , Canal, D. , & Potti, J. (2016). Nest‐dwelling ectoparasites reduce begging effort in Pied Flycatcher *Ficedula hypoleuca* nestlings. Ibis, 158(4), 881–886. 10.1111/ibi.12394

[ece38207-bib-0053] Naef‐Daenzer, B. , & Keller, L. F. (1999). The foraging performance of great and blue tits (*Parus major and P. caeruleus*) in relation to caterpillar development, and its consequences for nestling growth and fledging weight. Journal of Animal Ecology, 68(4), 708–718. 10.1046/j.1365-2656.1999.00318.x

[ece38207-bib-0054] Naef‐Daenzer, B. , Widmer, F. , & Nuber, M. (2001). Differential post‐fledging survival of great and coal tits in relation to their condition and fledging date. Journal of Animal Ecology, 70(5), 730–738. 10.1046/j.0021-8790.2001.00533.x

[ece38207-bib-0055] Nagy, L. R. , & Holmes, R. T. (2004). Factors influencing fecundity in migratory songbirds: Is nest predation the most important? Journal of Avian Biology, 35(6), 487–491. 10.1111/j.0908-8857.2004.03429.x

[ece38207-bib-0056] Nagy, L. R. , & Holmes, R. T. (2005). Food limits annual fecundity of a migratory songbird: An experimental study. Ecology, 86, 675–681. 10.1890/04-0155

[ece38207-bib-0057] Nieminen, P. , Lehtiniemi, H. , Vähäkangas, K. , Huusko, A. , & Rautio, A. (2013). Standardised regression coefficient as an effect size index in summarising findings in epidemiological studies. Epidemiology, Biostatistics and Public Health, 10, e8854‐1–e8854‐15. 10.2427/8854

[ece38207-bib-0058] Nord, A. , & Nilsson, J.‐Å. (2011). Incubation temperature affects growth and energy metabolism in Blue Tit nestlings. The American Naturalist, 178(5), 639–651. 10.1086/662172 22030733

[ece38207-bib-0059] Numata, M. , Davis, L. S. , & Renner, M. (2004). Growth and survival of chicks in relation to nest attendance patterns of little penguins (*Eudyptula minor*) at Oamaru and Motuara Island, New Zealand. New Zealand Journal of Zoology, 31(3), 263–269. 10.1080/03014223.2004.9518379

[ece38207-bib-0060] O’Brien, E. L. , & Dawson, R. D. (2008). Parasite‐mediated growth patterns and nutritional constraints in a cavity‐nesting bird. Journal of Animal Ecology, 77(1), 127–134. 10.1111/j.1365-2656.2007.01315.x 18177333

[ece38207-bib-0061] Oksanen, J. , Blanchet, F. G. , Friendly, M. , Kindt, R. , Legendre, P. , McGlinn, D. , Minchin, P. R. , O’Hara, R. B. , Simpson, G. L. , Solymos, P. , Stevens, M. H. H. , Szoecs, E. , & Wagner, H. (2019). *Vegan: Community Ecology Package* (R package version 2.5‐6) [Computer software]. https://CRAN.R‐project.org/package=vegan

[ece38207-bib-0062] Pérez, J. H. , Krause, J. S. , Chmura, H. E. , Bowman, S. , McGuigan, M. , Asmus, A. L. , Meddle, S. L. , Hunt, K. E. , Gough, L. , Boelman, N. T. , & Wingfield, J. C. (2016). Nestling growth rates in relation to food abundance and weather in the Arctic. The Auk, 133(2), 261–272. 10.1642/AUK-15-111.1

[ece38207-bib-0063] Petit, M. , & Vezina, F. (2014). Phenotype manipulations confirm the role of pectoral muscles and haematocrit in avian maximal thermogenic capacity. Journal of Experimental Biology, 217(6), 824–830. 10.1242/jeb.095703 24198261

[ece38207-bib-0064] Pinheiro, J. , Bates, D. , DebRoy, S. , Sarkar, D. , Eispack, S. , Heisterkamp, S. , Van Willigen, B. , & Ranke, J. (2021). nlme: Linear and Nonlinear Mixed Effects Models (3.1‐152) [Computer software]. https://CRAN.R‐project.org/package=nlme

[ece38207-bib-0065] Potti, J. , Moreno, J. , Merino, S. , Frías, O. , & Rodríguez, R. (1999). Environmental and genetic variation in the haematocrit of fledgling pied flycatchers *Ficedula hypoleuca* . Oecologia, 120(1), 1–8. 10.1007/s004420050826 28308040

[ece38207-bib-0066] R Core Team (2020). R: A language and environment for statistical computing. Vienna, Austria: R Foundation for Computing. https://www.R‐project.org/

[ece38207-bib-0067] Rauter, C. , Brodmann, P. , & Reyer, H. (2000). Provisioning behaviour in relation to food availability and nestling food demand in the Water Pipit *Anthus spinoletta* . Ardea, 88(1), 81–90.

[ece38207-bib-0068] Ronget, V. , Gaillard, J.‐M. , Coulson, T. , Garratt, M. , Gueyffier, F. , Lega, J.‐C. , & Lemaître, J.‐F. (2018). Causes and consequences of variation in offspring body mass: Meta‐analyses in birds and mammals. Biological Reviews, 93(1), 1–27. 10.1111/brv.12329 28393457

[ece38207-bib-0069] Saino, N. , Calza, S. , Møller, A. P. , & Moller, A. P. (1998). Effects of a dipteran ectoparasite on immune response and growth trade‐offs in barn swallow, *Hirundo rustica*, nestlings. Oikos, 81(2), 217–228. 10.2307/3547043

[ece38207-bib-0070] Salaberria, C. , Celis, P. , López‐Rull, I. , & Gil, D. (2014). Effects of temperature and nest heat exposure on nestling growth, dehydration and survival in a Mediterranean hole‐nesting passerine. Ibis, 156(2), 265–275. 10.1111/ibi.12121

[ece38207-bib-0072] Sih, A. (2013). Understanding variation in behavioural responses to human‐induced rapid environmental change: A conceptual overview. Animal Behaviour, 85(5), 1077–1088. 10.1016/j.anbehav.2013.02.017

[ece38207-bib-0073] Sih, A. , Stamps, J. , Yang, L. H. , McElreath, R. , & Ramenofsky, M. (2010). Behavior as a key component of integrative biology in a human‐altered world. Integrative and Comparative Biology, 50(6), 934–944. 10.1093/icb/icq148 21558249

[ece38207-bib-0074] Simon, A. , Thomas, D. W. , Speakman, J. R. , Blondel, J. , Perret, P. , & Lambrechts, M. M. (2005). Impact of ectoparasitic blowfly larvae (*Protocalliphora* spp.) on the behavior and energetics of nestling Blue Tits. Journal of Field Ornithology, 76(4), 402–410. 10.1648/0273-8570-76.4.402

[ece38207-bib-0075] Sofaer, H. R. , Chapman, P. L. , Sillett, T. S. , & Ghalambor, C. K. (2013). Advantages of nonlinear mixed models for fitting avian growth curves. Journal of Avian Biology, 44(5), 469–478. 10.1111/j.1600-048X.2013.05719.x

[ece38207-bib-0076] Stange, E. E. , Ayres, M. P. , & Bess, J. A. (2011). Concordant population dynamics of Lepidoptera herbivores in a forest ecosystem. Ecography, 34(5), 772–779. 10.1111/j.1600-0587.2010.06940.x

[ece38207-bib-0077] Tinbergen, J. M. , & Dietz, M. W. (1994). Parental energy expenditure during brood rearing in the Great Tit (*Parus major*) in relation to body mass, temperature, food availability and clutch size. Functional Ecology, 8(5), 563. 10.2307/2389916

[ece38207-bib-0078] Tremblay, I. , Thomas, D. , Blondel, J. , Perret, P. , & Lambrechts, M. M. (2005). The effect of habitat quality on foraging patterns, provisioning rate and nestling growth in Corsican Blue Tits *Parus caeruleus* . Ibis, 147(1), 17–24. 10.1111/j.1474-919x.2004.00312.x

[ece38207-bib-0079] Tripet, F. , Glaser, M. , & Richner, H. (2002). Behavioural responses to ectoparasites: Time‐budget adjustments and what matters to Blue Tits *Parus caeruleus* infested by fleas. Ibis, 144(3), 461–469. 10.1046/j.1474-919X.2002.00018.x

[ece38207-bib-0080] Tripet, F. , Richner, H. , & Tripet, F. (1997). Host responses to ectoparasites: Food compensation by parent Blue Tits. Oikos, 78(3), 557–561. 10.2307/3545617

[ece38207-bib-0081] Tuomainen, U. , & Candolin, U. (2011). Behavioural responses to human‐induced environmental change. Biological Reviews, 86(3), 640–657. 10.1111/j.1469-185X.2010.00164.x 20977599

[ece38207-bib-0082] Uller, T. , & Olsson, M. (2010). Offspring size and timing of hatching determine survival and reproductive output in a lizard. Oecologia, 162, 663–671. 10.1007/s00442-009-1503-x 19924446

[ece38207-bib-0083] Vafidis, J. O. , Facey, R. J. , Leech, D. , & Thomas, R. J. (2018). Supplemental food alters nest defence and incubation behaviour of an open‐nesting wetland songbird. Journal of Avian Biology, 49(8), e01672. 10.1111/jav.01672

[ece38207-bib-0084] Verhulst, S. , Perrins, C. M. , & Riddington, R. (1997). Natal dispersal of Great Tits in a patchy environment. Ecology, 78(3), 864–872.

[ece38207-bib-0085] Vleck, C. M. , & Priedkalns, J. (1985). Reproduction in Zebra Finches: Hormone levels and effect of dehydration. The Condor, 87(1), 37–46. 10.2307/1367129

[ece38207-bib-0086] Walker, M. D. , & Rotherham, I. D. (2011). No evidence of increased parental investment by Common Swifts *Apus apus* in response to parasite load in nests. Bird Study, 58(2), 217–220. 10.1080/00063657.2010.546391

[ece38207-bib-0087] Walker, R. E. , Keane, C. R. , & Burke, J. G. (2010). Disparities and access to healthy food in the United States: A review of food deserts literature. Health & Place, 16(5), 876–884. 10.1016/j.healthplace.2010.04.013 20462784

[ece38207-bib-0088] Webb, D. R. (1987). Thermal tolerance of avian embryos: A review. The Condor, 89(4), 874–898. 10.2307/1368537

[ece38207-bib-0089] Wegmann, M. , Voegeli, B. , & Richner, H. (2015). Physiological responses to increased brood size and ectoparasite infestation: Adult great tits favour self‐maintenance. Physiology & Behavior, 141, 127–134. 10.1016/j.physbeh.2015.01.017 25600467

[ece38207-bib-0090] Wiebe, K. L. , & Slagsvold, T. (2014). Prey size increases with nestling age: Are provisioning parents programmed or responding to cues from offspring? Behavioral Ecology and Sociobiology, 68(5), 711–719. 10.1007/s00265-014-1684-0

[ece38207-bib-0091] Williams, G. C. (1966). Natural selection, the costs of reproduction, and a refinement of Lack’s principle. The American Naturalist, 100(916), 687–690. 10.1086/282461

[ece38207-bib-0092] Williams, H. M. , & DeLeon, R. L. (2020). Using artificial intelligence classification of videos to examine the environmental, evolutionary and physiological constraints on provisioning behavior. Journal of Avian Biology, 51(8), 1–12. 10.1111/jav.02424

[ece38207-bib-0093] Williams, K. , Sudnick, M. , Anderson, R. , & Fitschen‐Brown, M. (2020). Experience counts: The role of female age in morning incubation and brooding behavior in relation to temperature. Journal of Avian Biology, 51(7), 1–12. 10.1111/jav.02397

[ece38207-bib-0099] Wolda, H. (1990). Food availability for an insectivore and how to measure it. Studies in Avian Biology, 13, 38–43.

[ece38207-bib-0094] Wong, B. , & Candolin, U. (2015). Behavioral responses to changing environments. Behavioral Ecology, 26(3), 665–673. 10.1093/beheco/aru183

[ece38207-bib-0095] Wright, J. , Both, C. , Cotton, P. A. , & Bryant, D. (1998). Quality vs. quantity: Energetic and nutritional trade‐offs in parental provisioning strategies. Journal of Animal Ecology, 67(4), 620–634. 10.1046/j.1365-2656.1998.00221.x

[ece38207-bib-0096] Yap, K. N. , Dick, M. F. , Guglielmo, C. G. , & Williams, T. D. (2018). Effects of experimental manipulation of hematocrit on avian flight performance in high‐ and low‐altitude conditions. Journal of Experimental Biology, 221(22), 1–10. 10.1242/jeb.191056 30266786

[ece38207-bib-0097] Zuur, A. , Ieno, E. N. , Walker, N. , Saveliev, A. A. , & Smith, G. M. (2009). Mixed effects models and extensions in ecology with R. Springer Science & Business Media.

