# Supplementary material for: Nature versus nurture: Structural equation modeling indicates that parental care does not mitigate consequences of poor environmental conditions in Eastern Bluebirds (Sialia sialis)
Source: Ecol Evol. 2021 Oct 20;11(21):15237–48. doi: 10.1002/ece3.8207 (PMC8571643; doi:10.1002/ece3.8207)
Supplement: Supplementary file 1 — Supplementary Material [file ECE3-11-15237-s002.pdf]

Supplemental Material: Structural Equation Models

S1. SEM with growth rate (K) as the response variable

```
fit.psem<-psem(
  lme(Growth~Food+Biomass+ bf + Prov+Att +Temp, random = ~1|Nest.ID, data=k.dat),
  lme(Prov~Food+Biomass+bf, random = ~1|Nest.ID, data=k.dat),
  lme(Att~Food+Biomass+bf, random = ~1|Nest.ID, data=k.dat),
  lme(Temp~Food+Biomass+bf, random = ~1|Nest.ID, data=k.dat)
  )

fit.psem1<-psem(
  lme(Growth~Food+Biomass+ bf + Prov+Att +Temp, random = ~1|Nest.ID, data=k.dat),
  lme(Prov~Food+Biomass+bf, random = ~1|Nest.ID, data=k.dat),
  lme(Att~Food+Biomass+bf, random = ~1|Nest.ID, data=k.dat),
  lme(Temp~Food+Biomass+bf, random = ~1|Nest.ID, data=k.dat),
  Prov%~~%Temp,
  Temp%~~%Brood,
  Att%~~%Prov,
  Prov%~~%Brood,
  Food%~~%Biomass
  )

fit.psem1.1<-psem(
  lme(Growth~Food+Biomass+ bf + Prov+Att +Temp, random = ~1|Nest.ID, data=k.dat),
  lme(Prov~Food+Biomass+bf, random = ~1|Nest.ID, data=k.dat),
  lme(Att~Food+Biomass+bf, random = ~1|Nest.ID, data=k.dat),
  lme(Temp~Food+Biomass+bf, random = ~1|Nest.ID, data=k.dat),
  Prov%~~%Temp,
  Temp%~~%Brood,
```

```
  Food%~~%Biomass
)

fit.psem2<-psem(
  lme(Growth~bf + Prov+Att +Temp, random = ~1|Nest.ID, data=k.dat),
  lme(Prov~Food+Biomass+bf, random = ~1|Nest.ID, data=k.dat),
  lme(Att~Food+Biomass+bf, random = ~1|Nest.ID, data=k.dat),
  lme(Temp~Food+Biomass+bf, random = ~1|Nest.ID, data=k.dat),
  Prov%~~%Temp,
  Temp%~~%Brood,
  Att%~~%Prov,
  Prov%~~%Brood,
  Food%~~%Biomass
  )

fit.psem3<-psem(
  lme(Growth~bf +Biomass+ Prov+Att +Temp, random = ~1|Nest.ID, data=k.dat),
  lme(Prov~Food+Biomass+bf, random = ~1|Nest.ID, data=k.dat),
  lme(Att~Food+Biomass+bf, random = ~1|Nest.ID, data=k.dat),
  lme(Temp~Food+Biomass+bf, random = ~1|Nest.ID, data=k.dat),
  Prov%~~%Temp,
  Temp%~~%Brood,
  Att%~~%Prov,
  Prov%~~%Brood,
  Food%~~%Biomass
)

fit.psem4<-psem(
```

```
  lme(Growth~bf +Biomass+ Prov+Att +Temp, random = ~1|Nest.ID, data=k.dat),
  lme(Prov~Food+Biomass+bf, random = ~1|Nest.ID, data=k.dat),
  lme(Att~Food+Biomass, random = ~1|Nest.ID, data=k.dat),
  lme(Temp~Food+Biomass+bf, random = ~1|Nest.ID, data=k.dat),
  Prov%~~%Temp,
  Temp%~~%Brood,
  Att%~~%Prov,
  Prov%~~%Brood,
  Food%~~%Biomass
)

# remove Att~Prov
fit.psem5<-psem(
  lme(Growth~bf +Biomass+ Prov+Att +Temp, random = ~1|Nest.ID, data=k.dat),
  lme(Prov~Food+Biomass+bf, random = ~1|Nest.ID, data=k.dat),
  lme(Att~Food+Biomass, random = ~1|Nest.ID, data=k.dat),
  lme(Temp~Food+Biomass+bf, random = ~1|Nest.ID, data=k.dat),
  Prov%~~%Temp,
  Temp%~~%Brood,
  Prov%~~%Brood,
  Food%~~%Biomass
)
#remove Growth~Att
fit.psem6<-psem(
  lme(Growth~bf +Biomass+ Prov+Temp, random = ~1|Nest.ID, data=k.dat),
  lme(Prov~Food+Biomass+bf, random = ~1|Nest.ID, data=k.dat),
  lme(Att~Food+Biomass, random = ~1|Nest.ID, data=k.dat),
  lme(Temp~Food+Biomass+bf, random = ~1|Nest.ID, data=k.dat),
```

```
  Prov%~~%Temp,
  Temp%~~%Brood,
  Att%~~%Prov,
  Prov%~~%Brood,
  Food%~~%Biomass
)
fit.psem4a<-psem(
  lme(Growth~bf +Prov+Att +Temp, random = ~1|Nest.ID, data=k.dat),
  lme(Prov~Food+Biomass+bf, random = ~1|Nest.ID, data=k.dat),
  lme(Att~Food+Biomass, random = ~1|Nest.ID, data=k.dat),
  lme(Temp~Food+Biomass+bf, random = ~1|Nest.ID, data=k.dat),
  Prov%~~%Temp,
  Temp%~~%Brood,
  Att%~~%Prov,
  Prov%~~%Brood,
  Food%~~%Biomass
)
```

S2. SEM with hematocrit as the response variable

```
fit.hem<-psem(
  lme(Hem~bf +Food + Biomass+ Prov+Att +Temp, random = ~1|Nest.ID, data=hem.dat),
  lme(Prov~Food+Biomass+bf, random = ~1|Nest.ID, data=hem.dat),
  lme(Att~Food+Biomass+bf, random = ~1|Nest.ID, data=hem.dat),
  lme(Temp~Food+Biomass+bf, random = ~1|Nest.ID, data=hem.dat))

fit.hem.psem<-psem(
  lme(Hem~bf +Food + Biomass+ Prov+Att +Temp, random = ~1|Nest.ID, data=hem.dat),
  lme(Prov~Food+Biomass+bf, random = ~1|Nest.ID, data=hem.dat),
  lme(Att~Food+Biomass+bf, random = ~1|Nest.ID, data=hem.dat),
  lme(Temp~Food+Biomass+bf, random = ~1|Nest.ID, data=hem.dat),
  Prov%~~%Temp,
  Temp%~~%Brood,
  Att%~~%Prov,
  Prov%~~%Brood,
  Food%~~%Biomass
)
fit.hem.psem1<-psem(
  lme(Hem~bf + Biomass+ Prov+Att +Temp, random = ~1|Nest.ID, data=hem.dat),
  lme(Prov~Biomass+bf, random = ~1|Nest.ID, data=hem.dat),
  lme(Att~Biomass+bf, random = ~1|Nest.ID, data=hem.dat),
  lme(Temp~Biomass+bf, random = ~1|Nest.ID, data=hem.dat),
  Prov%~~%Temp,
  Temp%~~%Brood,
  Att%~~%Prov,
  Prov%~~%Brood
)
```

```
summary(fit.hem.psem1) # aic 46.5 but I think I want to keep food # aic = 46.18, p = 92, df = 2
#use food instead of biomss
fisherC(fit.hem.psem1)
AIC(fit.hem.psem1)


fit.hem.psem1.1<-psem(
  lme(Hem~bf + Food+ Prov+Att +Temp, random = ~1|Nest.ID, data=hem.dat),
  lme(Prov~Food+bf, random = ~1|Nest.ID, data=hem.dat),
  lme(Att~Food+bf, random = ~1|Nest.ID, data=hem.dat),
  lme(Temp~Food+bf, random = ~1|Nest.ID, data=hem.dat),
  Prov%~~%Temp,
  Temp%~~%Brood,
  Att%~~%Prov,
  Prov%~~%Brood
)

fit.hem.psem2<-psem(
  lme(Hem~bf +Food + Biomass+ Prov+Att +Temp, random = ~1|Nest.ID, data=hem.dat),
  lme(Prov~Food+Biomass, random = ~1|Nest.ID, data=hem.dat),
  lme(Att~Food+Biomass, random = ~1|Nest.ID, data=hem.dat),
  lme(Temp~Food+Biomass, random = ~1|Nest.ID, data=hem.dat),
  Prov%~~%Temp,
  Temp%~~%Brood,
  Att%~~%Prov,
  Prov%~~%Brood,
  Food%~~%Biomass
)
```

```
fit.hem.psem2.1<-psem(
 lme(Hem~bf +Food + Biomass+ Prov, random = ~1|Nest.ID, data=hem.dat),
 lme(Prov~Food+Biomass, random = ~1|Nest.ID, data=hem.dat),
 lme(Att~Food+Biomass, random = ~1|Nest.ID, data=hem.dat),
 lme(Temp~Food+Biomass, random = ~1|Nest.ID, data=hem.dat),
 Prov%~~%Temp,
 Temp%~~%Brood,
 Att%~~%Prov,
 Prov%~~%Brood,
 Food%~~%Biomass

fit.hem.psem2.2<-psem(
 lme(Hem~bf + Biomass+ Prov, random = ~1|Nest.ID, data=hem.dat),
 lme(Prov~Food+Biomass, random = ~1|Nest.ID, data=hem.dat),
 lme(Att~Food+Biomass, random = ~1|Nest.ID, data=hem.dat),
 lme(Temp~Food+Biomass, random = ~1|Nest.ID, data=hem.dat),
 Prov%~~%Temp,
 Temp%~~%Brood,
 Att%~~%Prov,
 Prov%~~%Brood,
 Food%~~%Biomass
)

fit.hem.psem2.3<-psem(
 lme(Hem~ Biomass+ Prov, random = ~1|Nest.ID, data=hem.dat),
 lme(Prov~Food+Biomass, random = ~1|Nest.ID, data=hem.dat),
 lme(Att~Food+Biomass, random = ~1|Nest.ID, data=hem.dat),
```

```
  lme(Temp~Food+Biomass, random = ~1|Nest.ID, data=hem.dat),
  Prov%~~%Temp,
  Temp%~~%Brood,
  Att%~~%Prov,
  Prov%~~%Brood,
  Food%~~%Biomass
)

fit.hem.psem2.3.1<-psem(
  lme(Hem~ Biomass+ Prov, random = ~1|Nest.ID, data=hem.dat),
  lme(Prov~Food+Biomass, random = ~1|Nest.ID, data=hem.dat),
  lme(Att~Food+Biomass, random = ~1|Nest.ID, data=hem.dat),
  lme(Temp~Food+Biomass, random = ~1|Nest.ID, data=hem.dat),
  Prov%~~%Temp,
  Temp%~~%Brood,
  Att%~~%Prov,
  Prov%~~%Brood,
  Food%~~%Biomass,
  Temp%~~%Att
)
```
