# Supplementary material for: Nature versus nurture: Structural equation modeling indicates that parental care does not mitigate consequences of poor environmental conditions in Eastern Bluebirds (Sialia sialis)
Source: Ecol Evol. 2021 Oct 20;11(21):15237–48. doi: 10.1002/ece3.8207 (PMC8571643; doi:10.1002/ece3.8207)
Supplement: Supplementary file 2 — Supplementary Material [file ECE3-11-15237-s001.docx]

# Appendices

**Table S1.** Predictor variables included in models investigating growth rate in relation to provisioning rate, nest attendance, available arthropod biomass, volume of small arthropods (1-10mm), and presence of blow flies with nest ID as a random effect. Fixed effects, AICc, ΔAICc and the AICc model weights are presented.

| Fixed Effects | AICc | ΔAICc | weight |
| --- | --- | --- | --- |
| Blow flies + arthropod mass + volume small arthropods | 902.6 | 0 | 0.17 |
| Blow flies | 903.2 | 0.62 | 0.125 |
| Blow flies + arthropod mass | 903.3 | 0.7 | 0.12 |
| Blow flies+ volume small arthropods | 904 | 1.42 | 0.084 |
| Blow flies + arthropod mass+ volume small arthropods + provisioning rate | 904.7 | 2.05 | 0.061 |
| Attendance + blow flies + arthropod mass+ volume small arthropods | 904.7 | 2.06 | 0.061 |
| Attendance + blow flies | 905.3 | 2.66 | 0.045 |
| Blow flies + provisioning rate | 905.3 | 2.66 | 0.045 |
| Attendance + blow flies + arthropod mass | 905.4 | 2.75 | 0.043 |
| Blow flies + arthropod mass + provisioning rate | 905.4 | 2.76 | 0.043 |
| Blow flies + arthropod mass + provisioning rate | 905.4 | 2.76 | 0.043 |
| Attendance + blow flies+ volume small arthropods | 906.1 | 3.47 | 0.03 |
| Blow flies+ volume small arthropods + provisioning rate | 906.1 | 3.47 | 0.03 |
| Blow flies + arthropod mass+ volume small arthropods + provisioning rate + attendance*blow flies | 906.7 | 4.09 | 0.022 |
| Attendance + blow flies + provisioning rate | 907.3 | 4.71 | 0.016 |
| Attendance + blow flies + arthropod mass + attendance*blow flies | 907.4 | 4.8 | 0.015 |
| Attendance + blow flies + arthropod mass + provisioning rate | 907.4 | 4.81 | 0.015 |
| Attendance + blow flies+ volume small arthropods+ provisioning rate | 908.1 | 5.53 | 0.011 |
| Attendance + blow flies + arthropod mass + volume small arthropods + provisioning rate | 910.6 | 8.02 | 0.003 |
| Volume small arthropods | 910.9 | 8.34 | 0.003 |
| Arthropod mass + arthropod mass | 911.1 | 8.49 | 0.002 |
| Attendance | 911.6 | 8.95 | 0.002 |
| Provisioning rate | 911.6 | 8.96 | 0.002 |
| Arthropod mass + arthropod mass + volume small arthropods | 911.9 | 9.33 | 0.002 |
| Attendance + volume small arthropods | 913 | 10.37 | 0.001 |
| Volume small arthropods + provisioning rate | 913 | 10.38 | 0.001 |
| Attendance + arthropod mass | 913.1 | 10.52 | 0.001 |
| Arthropod mass + arthropod mass + provisioning rate | 913.1 | 10.53 | 0.001 |
| Attendance + provisioning rate | 913.6 | 11 | 0.001 |
| Attendance + arthropod mass + volume small arthropods | 914 | 11.37 | 0.001 |
| Arthropod mass + arthropod mass + volume small arthropods + provisioning rate | 914 | 11.39 | 0.001 |
| Attendance + volume small arthropods + provisioning rate | 915 | 12.42 | 0 |
| Attendance + arthropod mass + provisioning rate | 915.2 | 12.57 | 0 |
| Attendance + arthropod mass + volume small arthropods + provisioning rate | 916 | 13.43 | 0 |

**Table S2.** Predictor variables included in models investigating hematocrit in relation to provisioning rate, nest attendance, available arthropod biomass, volume of small arthropods (1-10mm), brood size, and presence of blow flies with nest ID as a random effect. Fixed effects, AICc, ΔAICc and the AICc model weights are presented.

| Fixed Effects | AICc | ΔAICc | weight |
| --- | --- | --- | --- |
| Brood size + blow flies + arthropod mass | 1142.2 | 0 | 0.169 |
| Brood size + blow flies + arthropod mass + volume small arthropods | 1142.6 | 0.4 | 0.138 |
| Brood size + arthropod mass | 1144 | 1.75 | 0.07 |
| Brood size + arthropod mass + volume small arthropods | 1144.1 | 1.93 | 0.064 |
| Brood size + blow flies + arthropod mass + provisioning rate | 1144.2 | 1.99 | 0.062 |
| Brood size + blow flies + arthropod mass + attendance | 1144.3 | 2.04 | 0.061 |
| Brood size + blow flies + arthropod mass + volume small arthropods + provisioning rate | 1144.6 | 2.39 | 0.051 |
| Brood size + blow flies + arthropod mass + volume small arthropods + attendance | 1144.7 | 2.45 | 0.05 |
| Brood size + blow flies | 1145 | 2.75 | 0.043 |
| Brood size + arthropod mass + provisioning rate | 1146 | 3.75 | 0.026 |
| Brood size + arthropod mass + attendance | 1146 | 3.78 | 0.025 |
| Brood size + arthropod mass + volume small arthropods + provisioning rate | 1146.1 | 3.93 | 0.024 |
| Brood size + arthropod mass + volume small arthropods + attendance | 1146.2 | 3.97 | 0.023 |
| Brood size + blow flies + arthropod mass + provisioning rate + attendance | 1146.3 | 4.04 | 0.022 |
| Brood size + blow flies + arthropod mass + attendance + attendance*blow flies | 1146.3 | 4.04 | 0.022 |
| Brood size + blow flies + arthropod mass + volume small arthropods + provisioning rate + provisioning * blow flies | 1146.6 | 4.43 | 0.018 |
| Brood size + blow flies + arthropod mass + volume small arthropods + provisioning rate + attendance | 1146.7 | 4.44 | 0.018 |
| Brood size + blow flies + volume small arthropods | 1146.7 | 4.48 | 0.018 |
| Brood size + blow flies + provisioning rate | 1147 | 4.76 | 0.016 |
| Brood size + blow flies + attendance | 1147 | 4.79 | 0.015 |
| Brood size + arthropod mass + provisioning rate + attendance | 1148 | 5.78 | 0.009 |
| Brood size + arthropod mass + volume small arthropods + provisioning rate + attendance | 1148.2 | 5.96 | 0.009 |
| Brood size + blow flies + volume small arthropods + provisioning rate | 1148.7 | 6.49 | 0.007 |
| Brood size + blow flies + volume small arthropods + attendance | 1148.7 | 6.52 | 0.006 |
| Brood size + volume small arthropods | 1149 | 6.78 | 0.006 |
| Brood size + blow flies + provisioning rate + attendance | 1149 | 6.8 | 0.006 |
| Brood size + provisioning rate | 1149.3 | 7.08 | 0.005 |
| Brood size + attendance | 1149.3 | 7.1 | 0.005 |
| Brood size + blow flies + arthropod mass + volume small arthropods + provisioning rate + attendance*blow flies + provisioning * blow flies | 1150.7 | 8.48 | 0.002 |
| Brood size + blow flies + volume small arthropods + provisioning rate + attendance | 1150.8 | 8.54 | 0.002 |
| Brood size + volume small arthropods + provisioning rate | 1151 | 8.79 | 0.002 |
| Brood size + volume small arthropods + attendance | 1151 | 8.81 | 0.002 |
| Brood size + provisioning rate + attendance | 1151.3 | 9.11 | 0.002 |
| Brood size + volume small arthropods + provisioning rate + attendance | 1153 | 10.83 | 0.001 |

| **Growth Rate** | AICc | ΔAICc | Weight |
| --- | --- | --- | --- |
| Incubation temperature | 955.2 | 0 | 0.424 |
| Brooding temperature | 955.2 | 0.09 | 0.406 |
| Brooding temperature + incubation temperature | 957 | 1.83 | 0.17 |

**Table S3.** Predictor variables included in models investigating growth rate in relation to incubation and brooding temperature with nest ID as a random effect. Fixed effects, AICc, ΔAICc and the AICc model weights are presented.

| **Hematocrit** | AICc | ΔAICc | Weight |
| --- | --- | --- | --- |
| Brood size + incubation temperature | 1087.6 | 0 | 0.514 |
| Brood size + brooding temperature | 1089.1 | 1.44 | 0.251 |
| Brood size + brooding temperature + incubation temperature | 1089.2 | 1.56 | 0.236 |

**Table S4.** Predictor variables included in models investigating hematocrit in relation to incubation and brooding temperature with nest ID as a random effect. Fixed effects, AICc, ΔAICc and the AICc model weights are presented.

Table S5. Model selection table for piecewiseSEM of growth rate (K) with Nest ID as a random effect. The full model without correlations (fit.psem) included the following response and fixed effects: Growth ~ Food + Biomass + bf + Prov + Att + Temp, Prov ~ Food +Biomass + bf, Att ~ Food +Biomass + bf, Temp ~ Food + Biomass + bf (all models are listed in Supplemental Material S1 and available in the code provided on GitHub <https://github.com/ecologykelly/SemEcoTutorial>). Model name, Fisher’s C, df, and p-value, AIC, Correlations included in the model. Directed sep. are relationships that were not in the model but needed based on tests of directed separation (+) or in model but not needed (-), Fixed effects added (+) or removed (-) from model compared to previous model. The AIC selected model is indicated in bold.

| Model | C (df, P) | AIC | Correlations | Directed sep. | Fixed effect |
| --- | --- | --- | --- | --- | --- |
| fit.psem | 44.95 (6, 0) | 98.946 | none | + Att ~ Prov  + Temp ~ Prov |  |
| fit.psem1 | 2.24 (2, 0.33) | 56.244 | Prov ~~ Temp  Temp ~~ Brood  Att ~~ Prov  Prov ~~ Brood  Food ~~ Biomass | - Att ~ Prov  - Prov ~ Brood |  |
| fit.psem1.1 | 7.15 (4, 0.13) | 61.152 | Prov ~~ Temp  Temp ~~ Brood  Food ~~ Biomass | + Att~Prov |  |
| fit.psem2 | 10.33 (6, 0.11) | 60.33 | Prov ~~ Temp Temp ~~ Brood Att ~~ Prov  Prov ~~ Brood  Food ~~ Biomass | + Growth ~ Biomass | - Growth ~ Biomass + Food |
| fit.psem3 | 3.93 (4, 0.42) | 55.93 | Prov ~~ Temp Temp ~~ Brood Att ~~ Prov  Prov ~~ Brood  Food ~~ Biomass |  | + Growth ~ Biomass |
| **fit.psem4** | **4.84 (6, 0.56)** | **54.84** | Prov ~~ Temp Temp ~~ Brood Att ~~ Prov  Prov ~~ Brood  Food ~~ Biomass |  | **- Att ~ bf** |
| fit.psem4a | 11.24 (8 ,0.19) | 59.24 | Prov ~~ Temp Temp ~~ Brood Att ~~ Prov  Prov ~~ Brood  Food ~~ Biomass | + Growth ~ Biomass |  |
| fit.psem5 | 9.75 (8, 0.28) | 59.75 | Prov ~~ Temp Temp ~~ Brood Prov ~~ Brood  Food ~~ Biomass | + Att ~ Prov |  |
| fit.psem6 | 7.249 (8, 0.51) | 55.249 | Prov ~~ Temp Temp ~~ Brood Att ~~ Prov  Prov ~~ Brood  Food ~~ Biomass |  | - Growth ~ Att |

Table S6. Model selection table for piecewiseSEM of hematocrit with Nest ID as a random effect. The full model without correlations included the following response and fixed effects: Hem ~ bf + Food + Biomass + Prov + Att + Temp, Prov ~ Food + Biomass + bf, Att ~ Food + Biomass + bf and Temp ~ Food + Biomass + bf (all models are listed in Supplemental Material S2 and available in the code provided on GitHub <https://github.com/ecologykelly/SemEcoTutorial>). Model name, Fisher’s C, df, and p-value, AIC, Correlations included in the model. Directed sep. are relationships that were not in the model but needed based on tests of directed separation (+) or in model but not needed (-), Fixed effects added (+) or removed (-) from model compared to previous model. The AIC selected model is indicated in bold.

| Model | C (df, P) | AIC | Correlations | Directed sep. | Fixed effect |
| --- | --- | --- | --- | --- | --- |
| fit.hem | 56.732 (6, 0) | 110.732 | none | + Att ~ Prov  + Temp ~ Prov  + Temp ~ Att |  |
| fit.hem.psem | 6.63 (2, 0.04) | 60.626 | Prov ~~ Temp  Temp ~~ Brood  Att ~~ Prov  Prov ~~ Brood Food ~~ Biomass | + Temp ~ Att |  |
| fit.hem.psem1 | 3.09 (2, 0.21) | 49.091 | Prov ~~ Temp  Temp ~~ Brood  Att ~~ Prov  Prov ~~ Brood | - Temp ~ Att | - Food |
| fit.hem.psem1.1 | 7.00 (2, 0.03) | 53.002 | Prov ~~ Temp  Temp ~~ Brood  Att ~~ Prov  Prov ~~ Brood | + Temp ~ Att | - Biomass  + Food |
| fit.hem.psem2 | 7.95, (8, 0.44) | 55.95 | Prov ~~ Temp  Temp ~~ Brood  Att ~~ Prov  Prov ~~ Brood  Food ~~ Biomass | - Prov ~ bf  - Att ~ bf  - Temp ~ bf  - Temp ~ Att | + Food & Biomass  - Att ~ bf  - Temp ~ bf  - Prov ~ bf |
| fit.hem.psem2.1 | 9.01(12, 0.70) | 53.009 | Prov ~~ Temp  Temp ~~ Brood  Att ~~ Prov  Prov ~~ Brood  Food ~~ Biomass | - Prov ~ bf  - Att ~ bf  - Temp ~ bf  - Temp ~ Att  - Hem ~ Att  - Hem ~ Temp | - Att + Temp |
| fit.hem.psem2.2 | 11.59 (14, 0.64) | 53.587 | Prov ~~ Temp  Temp ~~ Brood  Att ~~ Prov  Prov ~~ Brood  Food ~~ Biomass | - Prov ~ bf  - Att ~ bf  - Temp ~ bf  - Temp ~ Att  - Hem ~ Att  - Hem ~ Temp | - Hem ~ Food |
| fit.hem.psem2.3 | 10.63 (8, 0.22) | 50.633 | Prov ~~ Temp  Temp ~~ Brood  Att ~~ Prov  Prov ~~ Brood Food ~~ Biomass | - Hem ~ Food  + Temp ~ Att  - Hem ~ Att  - Hem ~ Temp | - Hem ~ BF |
| **fit.hem.psem2.3.1** | **4.88 (6, 0.56)** | **44.88** | Prov ~~ Temp  Temp ~~ Brood  Att ~~ Prov  Prov ~~ Brood  Food ~~ Biomass Temp ~~ Att |  |  |
